# Foodborne Infections and Mortality Associated With Expressed Breastmilk, Donated Breastmilk, and Infant Formula in High‐Income Countries: A Scoping Review of Peer‐Reviewed Evidence Cases

**DOI:** 10.1111/1541-4337.70282

**Published:** 2025-09-19

**Authors:** Chelsea S. Amenah‐James, Ellen W. Evans, Sophia Komninou

**Affiliations:** ^1^ Public Health Unit, School of Health and Social Care, Faculty of Medicine, Health and Life Sciences Swansea University Swansea UK; ^2^ ZERO2FIVE Food Industry Centre, Food & Drink Research Unit Cardiff Metropolitan University Cardiff UK

**Keywords:** bacterial contamination, breast milk, donor human milk, foodborne infection, infant feeding, infant mortality, powdered infant formula

## Abstract

Infant feeding influences infant growth, development, survival, and long‐term health. Maternal expressed breast milk (MEBM), donor human milk (DHM), and powdered infant formula (PIF) serve as alternatives when direct breastfeeding is not feasible. However, these alternatives are susceptible to microbial contamination, posing risks of infection and mortality. Despite concerns about foodborne infections in neonates, no comprehensive synthesis has examined infections and deaths directly linked to contaminated infant milk across different feeding methods. This scoping review examines infections and deaths associated with contaminated MEBM, DHM, and PIF in high‐income countries over the past 25 years, identifying key pathogens and sources of contamination. A systematic search on Medline, Scopus, and Embase identified a total of 6867 studies, of which 19 were selected, with an additional study from references. Data extracted for feeding type, pathogen, contamination source, and clinical outcomes. Among 175 cases, 55 led to systemic infections, including 13 deaths. *Cronobacter sakazakii* and *Pseudomonas aeruginosa* were the most frequently implicated pathogens in the published papers. PIF and DHM were more often linked to infections than MEBM. Besides intrinsic PIF contamination, hospital milk blenders and dishwashers were found to be positive for pathogens. DHM cases reported positive cultures of the nasogastric tubes, milk bank pasteurizers, and hospital bottle warmers. MEBM contamination was reported through breast pumps and hospital sinks and drains. This review highlights the need for enhanced hygiene protocols in handling infant milk. These findings inform clinical and public health policies aimed at minimizing infection risks associated with alternative infant feeding methods.

## 1 Introduction

Infant feeding is a subject of public health concern. It is justifiable, as infant feeding practices determine the early childhood growth and development (Berti and Socha 2023) and the long‐term health outcomes (Clayton et al. 2024). According to UNICEF (2016a), the health and survival of infants are affected by a combination of what they are fed and how they are fed. Breast milk is known to contain all the nutrients required for development and growth in early infancy (World Health Organization 2023b). Additionally, it contains infection‐protective elements such as oligosaccharides, lysozymes, lactoferrin, cytokines, and antibodies (Stepanovich and Donn 2022), which help to reduce infants’ susceptibility to common childhood diseases and enhance survival at this stage of life (World Health Organization 2023b). In the long term, breastfeeding is associated with a lowered risk of obesity in childhood (Liu et al. 2022) and reduced risk of Type 2 diabetes and cancer of the ovaries and the breasts in mothers (Walters et al. 2019). It has the potential to save more than 820,000 child deaths annually (UNICEF 2016b), and the global aggregated cost of not breastfeeding is estimated at nearly $350 billion (Walters et al. 2019). Consequently, UNICEF and WHO together recommend exclusive breastfeeding, which entails commencing breastfeeding from the first hour of life to the sixth month, initially without the addition of any liquid, solid, or water, and afterward, adding a complementary feed to the breastfeeding till the second year of life or for longer (World Health Organization 2023a, 2023b).

### 1.1 Barriers to breastfeeding

Despite global policies that promote exclusive breastfeeding, such as UNICEF's Baby Friendly Hospital Initiative, less than 50% of infants below 6 months of age globally are exclusively breastfed (World Health Organization 2023b). For example, in Australia, 37.5% of infants had been exclusively breastfed at 6 months of age (Australian Bureau of Statistics 2023), 25.4% in the US (Centers for Disease Control and Prevention 2025), and, according to the latest infant feeding survey, 1% in the UK (McAndrew et al. 2012). While a high proportion of women initiate breastfeeding, many opt for alternatives such as breastmilk substitute (powdered or ready‐to‐use formula milk) before the sixth month of life (Blackshaw et al. 2021) due to a variety of maternal‐related factors such as insufficient milk production, latch problems, maternal medical condition, and the need to return to work (Chang et al. 2019).

Besides maternal reasons, practically, an increasing number of infants cannot be directly breastfed due to medical conditions that necessitate separation from their mothers for medical care (Li et al. 2024). These are vulnerable infants—premature neonates, low birth weight, and very sick babies—and around 30 million of these are born globally each year (World Health Organization 2018), representing 10%–15% of live births. Of these, 13.4 million are premature infants (World Health Organization 2023c). They often have poor coordination of the sucking, swallowing, and breathing mechanisms (Dodrill 2011). When the feeding needs of those infants can be covered by the birth mother, maternal expressed breast milk (MEBM) is encouraged (McGuire et al. 2004). However, when this is not feasible, possibly due to a maternal medical condition or insufficient milk supply, alternatives such as Donor Human Milk (DHM) or Powdered Infant Formula (PIF) are opted for (Dong et al. 2022; Meek and Noble 2022).

### 1.2 Risk of Pathogens in Alternative Feeding Modes

Beyond caring for infants' nutritional requirements, these alternative feeding modes should also be safe. Regrettably, they have the risk of pathogenic contamination (Blackshaw et al. 2021), with the potential harm to the infants meant to benefit from their consumption.

#### 1.2.1 Maternal Expressed Breast Milk

This refers to a mother's milk that is expressed by hand or the use of a breast pump to feed her baby, usually with or without undergoing freezing and thawing, but often without pasteurization (Blackshaw et al. 2021; Picaud et al. 2018). In a neonatal intensive care unit (NICU) at the Suez Canal University Hospitals, Egypt, Gad et al. (2021) reported the contamination of 90% of manually expressed breastmilk samples collected between January and December 2019 under regulated conditions. *Staphylococcus aureus* was isolated from over 50% of the samples, and *Staphylococcus epidermidis* and *Enterobacter* were also identified. In addition, culturing of expressed breastmilk of mothers of premature infants in a US‐based NICU yielded *S. epidermidis* (42.4%), *Enterococcus faecalis* (11%), *Actinobacter* species (9.8%), *Escherichia coli* (0.6%), *Klebsiella* (3.5%), *Serratia marcescens* (1.9%), and Group B *Streptococcus* species (0.4%) (Schanler et al. 2011). Breast milk has two main pathogenic contamination routes: mother‐to‐child transmission (MTCT) and extrinsic contamination (Blackshaw et al. 2021). Practices such as expressing breastmilk with poorly washed hands, recontamination of hands after washing by touching tap handles to turn them off, and poor sterilization of feeding kits can extrinsically introduce pathogens into MEBM, while storing milk after 4 h of stay at room temperature can allow them to increase pathogens if initially present. (Blackshaw et al. 2021; Gad et al. 2021). The NHS (n.d.) stipulates refrigerating at 4°C or less for a maximum of 8 days.

#### 1.2.2 Donor Human Milk

DHM implies breast milk that is expressed and donated by another mother to a regulated milk bank, where it is stored and can be pasteurized or unpasteurized in cases where donor screening is considered adequate (Blackshaw et al. 2021). Hence, it shares similar potential contamination hazards as MEBM (Blackshaw et al. 2021). Although the non‐spore form of bacteria and viruses in DHM is eliminated by heat pasteurization (Wesolowska et al. 2019), DHM has been associated with *Bacillus cereus* pre‐ and post‐pasteurization (Lewin et al. 2019).

#### 1.2.3 Powdered Infant Formula

This is not a sterile product as often thought, as it is associated with inherent and acquired bacterial contamination (Blackshaw et al. 2021). Inherent contamination relates to pathogens’ introduction, such as *Cronobacter* and *Salmonella* species, during processing (Blackshaw et al. 2021; Crawley et al. 2022). To kill potentially present contaminants, the World Health Organization (2007) recommends reconstituting PIF with water boiled and cooled to a minimum of 70°C. Unfortunately, even in the UK, 85% of PIF reconstructing machines dispense water at a lower temperature than the ideal (Grant et al. 2024). Acquired contamination may occur during reconstitution due to poor hygiene of the preparer, quality of water, and storage conditions after reconstitution (Blackshaw et al. 2021).

### 1.3 Susceptibility to the Effects of Contaminated Infant Milk

All infants are at risk of the consequences of contaminated infant milk. However, preterm infants are at higher risk (Coleman et al. 2021). This is due to their undeveloped immune systems, the permeable nature of their gastrointestinal tracts, which facilitates the translocation of pathogens (Crawley et al. 2022), lack of immune protection conferred by maternal milk, and the disruption of gut microbiome balance in the case of PIF (Blackshaw et al. 2021). In comparison with the immune system of term babies, preterm infants have reduced neutrophils and monocytes and a weak ability to activate T lymphocytes due to poor cytokine production, which results in poor detection and response to the presence of pathogens (Melville and Moss 2013). A study revealed that it takes 3 years to get to an equal state of the immune system of term infants (Muraro et al. 2017).

Given their vulnerability, the safest of the three feeding modes should logically be emphasized when direct breastfeeding is impractical. Previous reviews have examined different aspects of these feeding options. Some reviews compared the three feeding methods based on the general risk of infection (not contamination‐associated) and mortality (Strobel et al. 2022). Some reviews examined infant‐acquired infection from breastfeeding and DHM, with a main focus on MTCT, and reported extensively on transmissible viruses (Blackshaw et al. 2021; Peters et al. 2016). Peters et al. (2016) also examined the effect of transport medium and storing temperatures on bacterial contamination of expressed breast milk and the current guidelines for effectively cleaning breast milk equipment. Although Blackshaw et al. (2021) extensively reviewed infections associated with PIF up until 2018, PIF is added to this review to create a basis for comparison with those associated with MEBM and DHM within the period under this review. To our knowledge, this is the first review of the actual cases of infant infections and deaths, not the risk of infection, arising specifically from contaminated infant milk, with emphasis on bacterial contaminants.

### 1.4 Aims and Objectives

This review aims to examine evidence of infant infections related to MEBM, PIF, and DHM contamination in high‐income countries in the last 25 years. It is limited to high‐income countries due to comparable healthcare standards and because the result of the review aims to improve policies and practices within this setting. The review will answer the following questions: How many cases of infant pathogenic colonization, infection, and deaths resulting from contaminated MEBM, PIF, and DHM were published in peer‐reviewed journals in the last 25 years? Are there differences in the number of cases associated with these infant feeding options? What are the common infection‐causing contaminants, and what are the sources of contamination?

Answering those questions will suggest which feeding practice is least hazardous and the aspect of infant milk handling that is highly prone to contamination, which will inform future interventions to improve infant feeding practices.

## 2 Methods

This scoping review was based on preestablished study eligibility criteria. It was confirmed by the chair of Swansea University's School of Health and Social Care Ethics Committee that further approval was not required owing to the nature of the study.

### 2.1 Eligibility Criteria

#### 2.1.1 Inclusion Criteria

As indicated in Table 1, eligible studies are those (a) related to the infant population; (b) studies on infection or death of any infant associated with contaminated MEBM, PIF, or DHM, as these are the exposures that are examined in this review; (c) studies from high‐income countries based on the World Bank Grouping of countries (The World Bank 2025) classification; (d) those with the following study design: case studies, case reports, randomized controlled trials (RCTs), and observational studies; (e) studies published in the English language, due to language barriers and financial constraints of engaging translation services; and (f) studies published between the years 2000 and 2024. The search was limited to the last 25 years to provide up‐to‐date evidence on the subject.

**TABLE 1:** Inclusion and exclusion criteria.

Inclusion criteria	Exclusion criteria
Studies related to infants Studies that reported cases of infants’ colonization, infection, or death following contaminated MEBM, PIF, or DHM ingestion Studies on bacterial contaminants Case studies, RCTs, Observational studies, and Case reports Publication years between 2000–2024 Studies published in the English language Peer‐reviewed studies Studies from high‐income countries	Studies that examine the effect of human milk fortifiers, prebiotics, or probiotics on infants' nutrition or ability to handle infections Articles that generally discuss pathogens related to MEBM, PIF, or DHM contamination without isolating the pathogen from infants' clinical samples Studies that report on infant infection traceable to sources other than the contamination of MEBM, PIF, or DHM Studies on non‐bacterial pathogens Simulation studies Studies on the prevention of transmission of infection from mothers’ milk to the infant. Non‐English articles Studies from low‐ and middle‐income countries Reviews on the subject Studies that examined adverse outcomes resulting from feeding infants with MEBM, PIF, or DHM, that are not related to pathogenic contamination, such as a protein allergy from cow's milk Studies related to mother‐to‐child breast milk‐based transmitted infections Studies with unclear sources of infant infection Studies in which the elimination of the pathogen causing the contamination involved the administration of antibiotic therapy to the mother

#### 2.1.2 Exclusion Criteria

Excluded studies include those (a) reporting isolated pathogens from MEBM, PIF, or DHM without actual infant infection; (b) reporting infant infection or death resulting from MTCT pathogens via direct breastmilk, because the focus of this review is pathogens acquired via extrinsic milk contamination; (c) studies in which antibiotic therapy is administered to the mother to eliminate the pathogen causing the contamination, as this action infers an MTCT consideration; (d) studies that reported on infant infection traceable to other sources other than the contamination of infant milk types that are examined in this review, such as raw goat milk ingestion. The details of the exclusion criteria are presented in Table 1.

### 2.2 Search Strategy

An initial pilot search was conducted, and reference lists of identified literature were searched for relevant keywords to be applied to the main search. After that, an advanced search was conducted on three databases: Medline via EBSCO, Scopus via Elsevier, and Embase via Elsevier on June 24, 2024. These databases were chosen due to their relevance to the topic and the fact that they are among the databases with a large number of peer‐reviewed articles.

The search terms were developed using the PEOS framework (Population: Infants; Exposures: MEBM, DHM, and PIF; Outcome: Infection and death; Study design: case reports, case studies, RCTs, and observational studies). Title and abstract phrase search terms were developed using truncation and double quotation marks for the synonyms. Given that there are three exposures in this review, a separate search was conducted for each exposure, which are PE_1_OS, where E_1_ is MEBM; PE_2_OS, where E_2_ is PIF; and PE_3_OS, where E_3_ is DHM. Results were limited to peer‐reviewed studies. Afterward, all the results articles were combined before the screening.The s

Furthermore, given their peculiarities, a separate search strategy was adopted for each database. Scopus database lacks subject headings; therefore, only a title/abstract search using search terms was conducted. On Medline, MeSH terms were combined with title and abstract search terms using Boolean operators “OR” for each concept and “AND” to connect the concepts in each PEOS group. Similarly, Emtree subject headings were combined with title, abstract, and keyword search terms on Embase. (See Appendix A in Supporting Information for the details of the search strategy).

### 2.3 Data Extraction

Data was extracted using a Microsoft Excel spreadsheet.

## 3 Results

### 3.1 Selection of Studies

**FIGURE 1:**
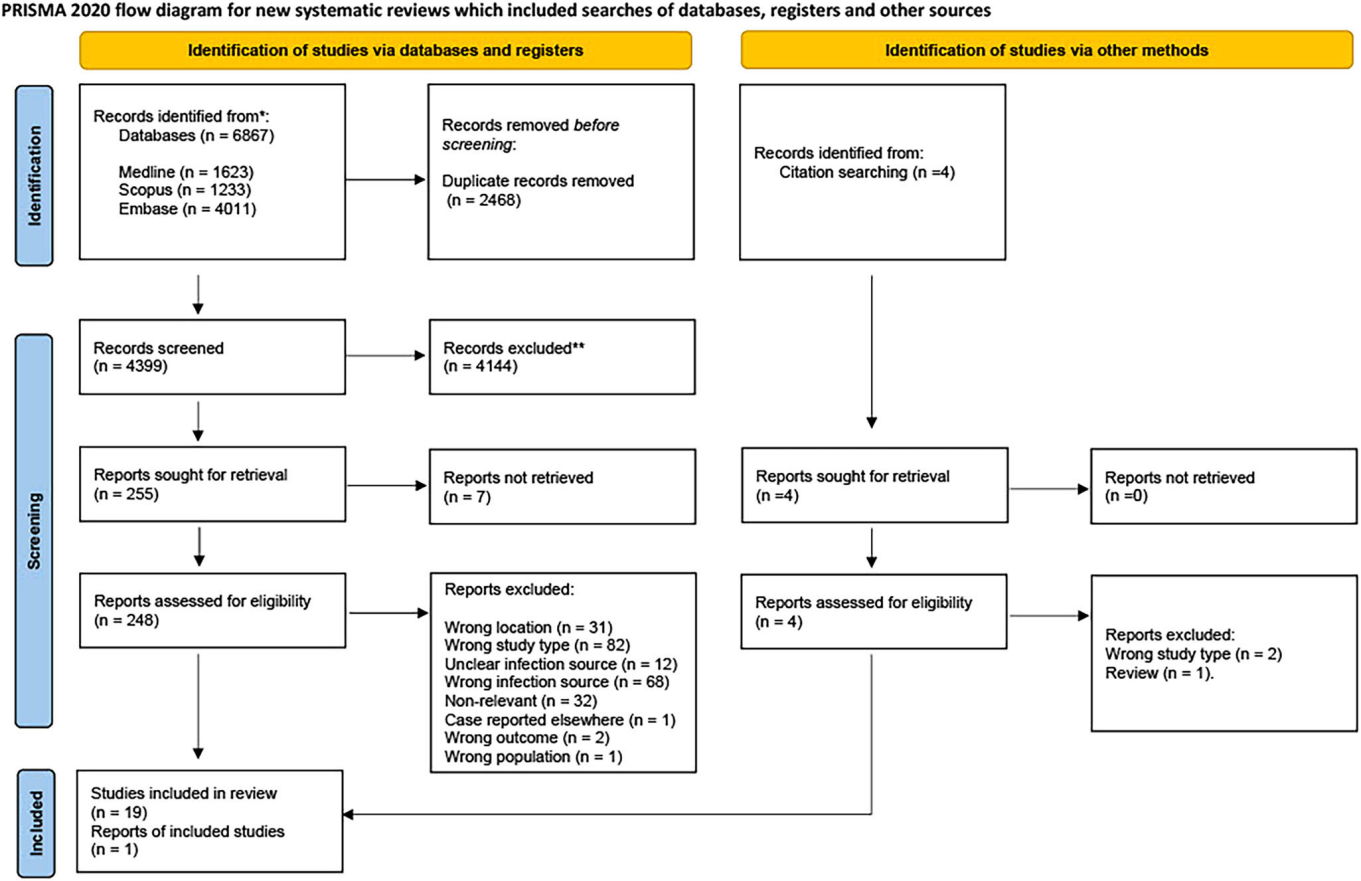
PRISMA 2020 flow diagram for new systematic reviews, which included searches of databases, registers, and other sources.

The database search yielded 6867 articles, which were exported to Covidence for the removal of duplicates and irrelevant studies in line with the exclusion criteria. One reviewer screened their titles and abstracts, and discussions were held with the second reviewer to resolve doubts. Thereafter, full‐text screening was conducted on the selected studies based on eligibility criteria, and 19 articles were found to be eligible. An additional study was retrieved from citation searching. Thus, 20 articles are included in this review. The reporting according to the Preferred Reporting Items for Systematic Reviews and Meta‐analysis (PRISMA) principles is shown in Figure 1.

### 3.2 Description of Studies

#### 3.2.1 Study Design

The 20 papers included in this review vary in study design but are mostly case reports. They consist of one cohort study (Himelright et al. 2002), one case‐control study (Bechmann et al. 2023), and 18 case reports and case series (Bar‐Oz et al. 2001; Bowen et al. 2017; Brett et al. 2005; Christoph et al. 2011; Decousser et al. 2013; Gras‐Le Guen et al. 2003; Liao and Tsai 2021; McMullan et al. 2018; Meeks et al. 2023; Mizuno et al. 2021; Ravisankar et al. 2014; Rettedal et al. 2012; Sánchez‐Carrillo et al. 2009; Smith and Serke 2016; Sundararajan et al. 2018; Taylor et al. 2021; Teramoto et al. 2010; Widger et al. 2010). Of these papers, four reported outbreaks from contaminated infant milk in NICUs (Bechmann et al. 2023; Gras‐Le Guen et al. 2003; Rettedal et al. 2012; Sánchez‐Carrillo et al. 2009).

#### 3.2.2 Methodological Quality Assessment

The risk of bias in the included articles was assessed using the JBI checklist for the observational studies (details in Appendices B and C in Supporting Information), while for the case reports, the published modified Newcastle–Ottawa Scale (as shown in Appendix D in Supporting Information) (Murad et al. 2018) was applied, as it was found more relevant and has been used in a systematic review involving the synthesis of evidence mainly from case reports, similar to this review (Stark et al. 2021). Half of the papers were appraised as of high quality (Bar‐Oz et al. 2001; Bechmann et al. 2023; Bowen et al. 2017; Brett et al. 2005; Decousser et al. 2013; Gras‐Le Guen et al. 2003; Himelright et al. 2002; Mizuno et al. 2021; Rettedal et al. 2012; Sánchez‐Carrillo et al. 2009), 45% as moderate (Christoph et al. 2011; Liao and Tsai 2021; McMullan et al. 2018; Meekset al. 2023; Ravisankar et al. 2014; Smith and Serke 2016; Sundararajan et al. 2018; Taylor et al. 2021; Widger et al. 2010), and 5% as weak in quality (Teramoto et al. 2010). The moderate‐ to low‐scoring studies failed to provide enough details on the cases and did not adequately explore confounding factors. (See Supporting Information for details of Quality Assessment).

#### 3.2.3 Description of the Exposures

Since this review examines cases of contaminated food related to three feeding methods, the articles are categorized accordingly. A total of 10 articles reported cases related to MEBM‐fed infants (Bowen et al. 2017; Christoph et al. 2011; Liao and Tsai 2021; McMullan et al. 2018; Mizuno et al. 2021; Ravisankar et al. 2014; Rettedal et al. 2012; Smith and Serke 2016; Sundararajan et al. 2018; Widger et al. 2010). Although Bowen et al. (2017) presented a mixed feeding case, it is grouped under the MEBM category because the contamination was traced to MEBM. Six papers presented cases of PIF contamination (Bar‐Oz et al. 2001; Brett et al. 2005; Himelright et al. 2002; Meeks et al. 2023; Sánchez‐Carrillo et al. 2009; Taylor et al. 2021), three described DHM‐related cases (Bechmann et al. 2023; Decousser et al. 2013; Gras‐Le Guen et al. 2003), and one of the reports presented a case of mixed feeding with MEBM, PIF, and milk additive (Teramoto et al. 2010). This case is treated separately because the authors failed to narrow the contamination to a particular feed. Categorizing the articles this way will provide deeper insight into the subject.

#### 3.2.4 Location

There were seven articles from the United States (Bowen et al. 2017; Himelright et al. 2002; Meeks et al. 2023; Ravisankar et al. 2014; Smith and Serke 2016; Sundararajan et al. 2018; Taylor et al. 2021), two from France (Decousser et al. 2013; Gras‐Le Guen et al. 2003) and Japan (Mizuno et al. 2021) (Teramoto et al. 2010), and one from Germany (Bechmann et al. 2023), Spain (Sánchez‐Carrillo et al. 2009), Israel (Bar‐Oz et al. 2001), Taiwan (Liao and Tsai 2021), Norway (Rettedal et al. 2012), Ireland (Widger et al. 2010), Australia (McMullan et al. 2018), Austria (Christoph et al. 2011), and the United Kingdom (Brett et al. 2005). Regarding the setting, 75% were NICU‐based and 25% were home‐based.

#### 3.2.5 Extent of Contaminant Source Investigation

Homogeneity is lacking in the extent of investigations conducted and the criteria used by the different authors to establish the source of the implicated organism. On one end of the spectrum, 12 reports were quite detailed in their investigations. On the other end, two reported no source investigation (Meeks et al. 2023; Teramoto et al. 2010).

### 3.3 Result Overview

A total of 175 cases resulting from contaminated MEBM, PIF, and DHM were reported. Of these, 135 were connected to outbreaks. Two outbreaks of *Pseudomonas aeruginosa* (Gras‐Le Guen et al. 2003; Sánchez‐Carrillo et al. 2009) and an outbreak of extended‐spectrum beta‐lactamase (ESBL)‐producing *Klebsiella pneumoniae* (Rettedal et al. 2012) and *S. marcescens* (Bechmann et al. 2023) were presented. Of the total cases, 120 were categorized as pathogenic colonization. This refers to cases in which the affected infants did not manifest symptoms, but the pathogenic organism was recovered from samples taken from the patient's non‐sterile body sites, such as the rectum (Rettedal et al. 2012). Fifty‐five (55) were outright cases of systemic infection in which affected neonates showed signs and symptoms and the bacteria was isolated from their sterile body sites such as the bloodstream or cerebrospinal fluid, with 13 of these resulting in death, which represents 24%, of which 46% of this was linked to *P. aeruginosa* contamination, 38% to *C. sakazakii* infection, and *E. coli* and *S. marcescens* were responsible for about 8% each.

Of the 55 infected cases, the birth category of 50 infants was reported. Out of these, a significant proportion (72%) were preterm, while only 28% were non‐preterm infants. Of the preterms whose gestational ages were indicated, half were ≤28 weeks, while the other half were >28 weeks but <37 weeks. Among the infected infants, the gender of about 50% of the cases was not reported. However, from the available reports, a male‐to‐female ratio of 10:19 was observed.

To appreciate the rate of contamination associated with each feeding practice, an in‐depth analysis of these incidents as they relate to MEBM, PIF, and DHM will now be discussed separately. Due to the heterogeneity of the exposures, organisms detected, investigations conducted, and the context, that is, a single case or an outbreak, a narrative synthesis of each infant food exposure along the lines of implicated pathogens and key learnings will be adopted in this review.

### 3.4 Infections Associated With MEBM

As illustrated in Table 2, the 10 studies that examined MEBM contamination presented an aggregate of 71 cases, consisting of 58 cases of colonization and 13 cases of infection, 2 of which culminated in death. Six foodborne pathogens were reported in contaminated MEBM‐related incidents. More studies (4) reported on *C. sakazakii* than any other pathogen (Bowen et al. 2017; McMullan et al. 2018; Ravisankar et al. 2014; Sundararajan et al. 2018). Two studies each identified *Klebsiella* (Rettedal et al. 2012; Widger et al. 2010), *P. aeruginosa* (Mizuno et al. 2021; Smith and Serke 2016), and *E. coli* (Christoph et al. 2011; Widger et al. 2010), and one paper each presented *B. cereus* (Liao and Tsai 2021) and methicillin‐resistant *S. aureus* (Smith and Serke 2016).

**TABLE 2:** Maternal expressed breast milk‐associated infections in high‐income countries, 2000–2014.

Authors and Country	Feed	Bacteria	Total cases	No colonized	No infected	No of deaths	Diagnosis of infected cases	M:F of infected cases	GA of infected cases (weeks + days)	Birth category of infected cases	BW of infected cases (g)
Bowen et al. (2017) USA	MEBM + DHM + fortifier	*C. sakazakii*	1	0	1	0	Severe meningitis	F	29	Preterm	1405
Christoph et al. (2011) Austria	MEBM	*E. coli*	2	0	2	0	Sepsis	NR	Twins 25+4	Preterm	850,767
Liao and Tsai (2021) Taiwan	MEBM via NGT	*B. cereus*	1	0	1	0	Bacteraemia	F	33+2	Preterm	1490
McMullan et al. (2018) Australia	MEBM via OGT	*C. sakazakii*	1	0	1	1	Meningitis	M	27+5	Preterm	NR
Mizuno et al. (2021) Japan	MEBM via FB	*P. aeruginosa*	1	0	1	0	Meningitis	F	40+3	Term	3436
Ravisankar et al. (2014) USA	MEBM via NGT	*C. sakazakii*	1	0	1	0	Meningitis	NR	27	Preterm	1140
Rettedal et al. (2012) Norway	MEBM	ESBL *Klebsiella*	57	57	0	0	NA	NA	NA	NA	NA
Smith and Serke (2016) USA	MEBM via OGT	Case 1*‐P. aeruginosa* Case 2 and 3 Twins*‐MRSA*	3	1	2	0	Case 1‐Sepsis Case 2‐Arm pustule	2M	Case 1‐25+2 Case 2–24	Preterm	Case 1‐830 Case 2‐570
Sundararajan et al. (2018) USA	MEBM	*C. sakazakii*	1	0	1	0	Meningitis	F	37	Term	NR
Widger et al. (2010) Ireland	MEBM	Twins*‐E. coli* Case 3‐*Klebsiella spp*.	3	0	3	1 (twin 1)	Sepsis	1 M, 2F	Twins‐32 Case 3‐25	Preterm	Twins 1 and 2 resp. 1020, 1390 Case 3. 790
Subtotal			71	585.	13	2					

Abbreviations: BW, birth weight; ESBL, extended‐spectrum beta, lactamase; FB, feeding bottle; GA, gestational age; MRSA, methicillin‐resistant *staphylococcus aureus*; NA, not applicable; NGT, nasogastric tube; NR, not reported; OGT, orogastric tube.

#### 3.4.1 Cronobacter sakazakii

The four cases of *C. sakazakii* accounted for nearly a third of all the cases of infection associated with this feeding alternative (Bowen et al. 2017; McMullan et al. 2018; Ravisankar et al. 2014; Sundararajan et al. 2018). Three out of these cases were reported from the United States (Bowen et al. 2017; Ravisankar et al. 2014; Sundararajan et al. 2018) and only one from another country, which was Australia (McMullan et al. 2018). Notably, from the reported data, 75% of these cases involved preterm patients whose gestational age ranged between 27 and 29 weeks and whose birth weights were less than 1500 g. More significant, though, is the fact that all four patients, both term and preterm infants, developed severe meningitis, and none had a mild infection. Severe brain affectation was evidenced by varying degrees of abnormal radiological findings such as leptomeningeal changes with multifocal diffusion (Sundararajan et al. 2018), periventricular hemorrhage, caseous necrosis of the brain tissue, cerebral oedema (Ravisankar et al. 2014), and unilateral frontal lobe and cerebral hemispheric liquefaction necrosis (Bowen et al. 2017), which necessitated ventriculoperitoneal shunt operation in two of the cases (Bowen et al. 2017; Ravisankar et al. 2014) and resulted in one death (McMullan et al. 2018).

A strength of three of these reports is that the authors eliminated confounders. Bowen et al. (2017), Sundararajan et al. (2018), and Ravisankar et al. (2014) confirmed that the reported cases had no previous history of PIF feeding, and Ravisankar et al. (2014) and Sundararajan et al. (2018) went further to report a negative history of the use of fortifiers before the onset of symptoms. Although the patient reported by Bowen et al. (2017) had mixed feeding of MEBM, DHM, and fortifiers, the authors demonstrated strong evidence for MEBM contamination through a positive culture result of the MEBM and a negative culture of the fortifier used. In contrast, McMullan et al. (2018) failed to explore such confounders, which is a limitation in their report.

An interesting highlight is seen in the culture results of the MEBM samples. Three groups of authors reported positive MEBM culture, which might appear to explain the source of infection (Bowen et al. 2017; McMullan et al. 2018; Sundararajan et al. 2018). On the contrary, in the report presented by Ravisankar et al. (2014), the MEBM sample tested negative for the organism isolated in the patient. At first glance, it can be argued that this discounts MEBM contamination as the origin of the infectious pathogen. However, the possibility of MEBM contamination is not unlikely given that the authors confirmed the patient's history of exclusive breastmilk feeding via nasogastric tube and excluded the use of fortifiers and PIF. A similar case reported by Decousser et al. (2013) under the contaminated DHM category demonstrates that the sterility of the milk does not eliminate the possibility of contamination, as contamination can occur from the last point at which the milk is introduced into the infant, which is the feeding tube. Ravisankar et al. (2014), however, failed to consider this possibility. Their study might have been more revealing if they had carried out a culture of the milk in the nasogastric tubes, as conducted by Decousser et al. (2013).

#### 3.4.2 Escherichia coli

Unlike contaminations with *C. sakazakii*, which were linked to severe infections in all four reported cases, contamination with *E. coli* varied in its clinical presentations. For instance, Christoph et al. (2011) reported an *E. coli* contamination that resulted in sepsis in a set of preterm twins delivered at 25 weeks and 4 days with VLBW of 850 and 767 g. Both recovered and were discharged. Remarkably, both infants were infected by the same pathogen even though they were managed from birth in two different wards, where there were no other cases of *E. coli*. This tends to support the authors’ argument that sepsis resulted from contaminated MEBM. By comparison, in a separate case report, sepsis from *E. coli* contamination involving a set of older (32 weeks) preterm twins, with higher birth weights (1020 and 1390 g), resulted in a deterioration of neurological function, intraventricular hemorrhage, and death of one of the twins—the twin with the lower birth weight (Widger et al. 2010).

#### 3.4.3 Klebsiella spp. 

*Klebsiella* species contamination of MEBM was reported in association with an outbreak in Norway (Rettedal et al. 2012) but in connection with a single case in Ireland (Widger et al. 2010). Rettedal et al. (2012) presented the country's first ESBL‐producing *K. pneumoniae* outbreak. The index case in this report had a systemic infection that was believed to have resulted from vertical transmission from a colonized mother, even though the maternal screening was not reported (the case is not included in this review due to the attributed mode of transmission). However, the 57 colonized infants are included in this review because these cases were attributed to hygiene compromise, as suggested by a positive culture of 60 samples, including the breast milk of three other mothers, a breast pump handle, and many hospital sink drains (Rettedal et al. 2012). Molecular analysis using pulse field gel electrophoresis also confirmed that 58 isolates out of these *Klebsiella*‐positive samples had the same strain as the clinical isolates (Rettedal et al. 2012). While this demonstrates that pathogens isolated in most of the colonized cases were sourced from the plausible index case, information on the owner of the pathogen‐positive breast pump and whether it is privately used by the mother of the index case or shared by other mothers in the unit could have provided further evidence of how the pathogen contaminated other mothers’ milk; however, the authors did not include this detail. Widger et al. (2010), on the other hand, highlighted the case of a 790 g female 25‐weeker who developed symptoms of sepsis secondary to *Klebsiella* infection 3 days after exposure to MEBM, and the mother had no prior history of mastitis. The MEBM, however, tested positive for the pathogen and shared the same antibiotic susceptibility as the strain isolated from the infant. The patient fully recovered. Unlike with *C. sakazakii*, severe infections or fatalities were not reported.

#### 3.4.4 *Bascillus cereus*, MRSA, and *Pseudomonas aeruginosa*

A case of bacteremia from contamination by *B. cereus* was reported in a female term infant by Liao and Tsai (2021). Smith and Serke (2016) documented a methicillin‐resistant *S. aureus*‐contaminated MEBM, which manifested as an arm pustule in a very low birth weight (VLBW) preterm. Mizuno et al. (2021) and Smith and Serke (2016) respectively highlighted cases of sepsis and meningitis that originated from *P. aeruginosa* infection. Although the neonate with meningitis developed a hearing disability, none of these cases was associated with death.

### 3.5 Infections Associated With PIF

As summarized in Table 3, the six authors who reported incidents associated with PIF contamination presented a total of 53 cases, which included 30 cases of pathogenic colonization and 23 cases of infections, resulting in five deaths.

**TABLE 3:** Powered infant formula milk‐associated infections in high‐income countries, 2000–2014.

Authors and Country	Feed	Bacteria	Total cases	No colonized	No infected	No of deaths	Diagnosis of infected cases	M:F of infected cases	GA of infected cases (weeks + days)	Birth category of infected cases	BW of infected cases (g)
Bar‐Oz et al. (2001) Israel	PIF via enteral tubes	*C. sakazakii*	5	3	2	0	Case 1‐Meningitis Case 2‐Bleeding tendency	2F	36 & 27 resp.	Preterm	2125 and 620 resp.
Brett et al. (2005) UK	PIF admin at home	*C. botulinum*	1	0	1	0	Infant botulism	F	5 months old	NA	NA
Himelright et al. (2002) USA	PIF via continuous administration	*C. sakazakii*	10	9	1	1	Meningitis	NR	33+5	Preterm	1270
Meeks et al. (2023) USA	PIF admin at home	*C. sakazakii*	5	0	5	2	Case 1‐Meningitis Others‐NR	NR	Case 1‐39	Case 1‐Term, Others NR	NR
Sánchez‐Carrillo et al. 2009 Spain	PIF via feeding bottles	*P. aeruginosa*	30	18	12	2	1 Peritonitis 11 Bacteraemia	5 M, 7F	NR	9 Preterm Others‐NR	≤1400
Taylor et al. (2021) USA	PIF admin at home	*C. sakazakii*	2	0	2	0	Case 1 and 2‐Meningitis	1 M, NR	NR	Term	NR
Subtotal			53	30	23	5					

Abbreviations: BW, birth weight; GA, gestational age; NA, not applicable; NR, not reported.

Three organisms were identified from these cases, namely, *P. aeruginosa* (Sánchez‐Carrillo et al. 2009), *C. sakazakii* (Bar‐Oz et al. 2001; Himelright et al. 2002; Meeks et al. 2023; Taylor et al. 2021), and *Clostridium botulinum* (Brett et al. 2005), with *C. sakazakii* being more reported than the others.

#### 3.5.1 Cronobacter sakazakii

As observed under MEBM contamination, *C. sakazakii* is also the most frequently reported pathogen in cases related to PIF contamination. Four out of the six papers reported on *C. sakazakii* (Bar‐Oz et al. 2001; Himelright et al. 2002; Meeks et al. 2023; Taylor et al. 2021), which was responsible for 43% of the cases of illnesses reported and caused 60% of the deaths under this classification. Similar to the picture seen among MEBM‐fed infant infections, *C. sakazakii* infections resulting from PIF contamination affected both term and preterm infants, and remarkably, almost all these cases with full clinical details, except one, manifested as meningitis.

#### 3.5.2 Pseudomonas aeruginosa

Unlike the single case of *P. aeruginosa* contamination reported under MEBM, an outbreak was detected, following routine noninvasive surveillance, in an NICU that practiced milk preparation using multidose PIF bottles (Sánchez‐Carrillo et al. 2009). According to the authors, a sudden increase from 1.9 to 8.8 cases per thousand was observed. A total of 18 neonates became colonized, 12 were infected, 1 of which developed peritonitis, and 2 died. The infected neonates were below 1400 g in weight and were all on exclusive PIF via feeding bottles (Sánchez‐Carrillo et al. 2009). Although 172 neonates were nursed in the ward during the outbreak period, the weight characteristics and feeding modalities for the unaffected children were not reported by the authors. This is important, as it might give an insight into the likely predisposing factors for the affected children.

#### 3.5.3 Clostridium botulinum

Brett et al. (2005) were the only authors who reported *C. botulinum* in association with PIF feeding. The patient was a 5‐month‐old female in the United Kingdom who presented with days of deteriorating neurological state, constipation, and a history of PIF feeding. Feces and rectal washout yielded *C. botulinum* type B and its neurotoxin. An open can of PIF tested positive for the same organism, with similar molecular patterns to those isolated from the child. Also, *C. botulinum* type B, but with a different pattern of DNA fingerprinting, was recovered from the unopened cans of PIF, which belonged to the same batch as the opened can. A diagnosis of infant botulism was made. Although the patient survived, she developed severe neurological sequelae. According to the authors, this was the first case of infant botulism in the United Kingdom to be traced to a likely source—intrinsically contaminated PIF.

### 3.6 Infections Associated With DHM

As observed in Table 4, 50 cases consisting of 32 colonized and 18 infected neonates, leading to five deaths, were reported by the three papers that presented DHM‐related incidents. Two of the reports were from France (Decousser et al. 2013; Gras‐Le Guen et al. 2003) and one from Germany (Bechmann et al. 2023). Each of the three clinical facilities described in these cases was supported by a milk bank, one of which supplied single‐donor breast milk (Bechmann et al. 2023), making it possible to trace the donor's identity of every milk portion the patients were fed, while another supplied pooled DHM (Decousser et al. 2013). The type of milk provided by the third milk bank was not reported (Gras‐Le Guen et al. 2003).

**TABLE 4:** Donor human milk‐associated infections in high‐income countries, 2000–2014.

Authors and country	Feed	Bacteria	Total cases	No colonized	No infected	No of deaths	Diagnosis of infected cases	M:F of infected cases	GA of infected cases (weeks + days)	Birth category of infected cases	BW of infected cases (g)
Bechmann et al. (2023) Germany	Single Donor DHM	*S. marcescens*	17	15	2	1	Case 1‐Meningitis Case 2‐Sepsis	2F	Case 1–29+2 Case 2‐NR	Case 1‐Preterm Case 2‐NR	Cases 1–1220 Cases 2–1230
Decousser et al. (2013) France	Pooled DHM via gastric tubes	*B. cereus*	2	0	2	0	Case 1‐SIIE Case 2‐SIIE+P	NR	Case 1–29 Case 2‐30	Preterm	Case 1–960 Case 2–1500
Gras‐Le Guen et al. (2003) France	DHM	*P*. *aeruginosa*	31	17	14	4	4 Fulminant septicemia 6 respiratory diseases 4 otitis media	NR	NFD	nine Preterm five Term	NFD
Subtotal			50	32	18	5					

Abbreviations: BW, birth weight; GA, gestational age; NA, not applicable; NFD, no full detail; NR, not reported; SIIE, severe intestinal infection and enterocolitis; SIIE+P, severe intestinal infection, enterocolitis with intestinal perforation.

*P. aeruginosa* (Gras‐Le Guen et al. 2003), *S. marcescens* (Bechmann et al. 2023), and *B. cereus* (Decousser et al. 2013) were identified in these cases. It is difficult to infer the commonest pathogen in this feeding practice, as few articles relating to it were found. Unlike in MEBM and PIF contamination, *C. sakazakii* was not reported.

#### 3.6.1 Serratia marcescens

Similar to Sánchez‐Carrillo et al.’s (2009) context, the outbreak of *S. marcescens* was detected following a routine weekly surveillance screening in an NICU, resulting in 15 colonized and two infected neonates, one of whom developed severe brain damage and died (Bechmann et al. 2023). It is significant that the suspected DHM in this outbreak, collected from a single donor, was culture‐negative at the time it was fed to the neonates. Hence, it was fed in an unpasteurized state. The negative culture result might seem to have excluded it as the source. The records, however, indicated that 7 out of 17 affected children were fed from the suspected DHM, and the donor had a previous history of *S. marcescens* culture‐positive milk samples, which were discarded according to milk bank guidelines. The other cases shared the same room and refrigerator for milk storage as the infected cases. Thus, the pathogen was speculated to have been transmitted by inadequate hygiene practices or secondary milk contamination (Bechmann et al. 2023). Confirmation of strain similarity was performed, which is one of the strengths of this report (Bechmann et al. 2023). A plausible explanation by the authors is the possibility that the fed milk contained a very low but viable *S. marcescens* concentration, which passed undetected by the culture method used, which could only detect concentrations at ≥100 CFU/mL.

#### 3.6.2 Pseudomonas aeruginosa

Similar to the *P. aeruginosa* outbreak described under contaminated PIF, which recorded 30, 18, 12, and 2 total cases, colonized, infected, and deaths, respectively (Sánchez‐Carrillo et al. 2009), the *P. aeruginosa* outbreak under contaminated DHM grouping reported 31, 17, 14, and 4 in the same order (Gras‐Le Guen et al. 2003). The 12 infected infants reported by Sánchez‐Carrillo et al. (2009) developed bacteremia, which was complicated by peritonitis in one of the cases, while Gras‐Le Guen et al. (2003) presented four cases of fulminant septicemia and six severe respiratory infections in VLBW preterm neonates, and four cases of otitis media in the term infants (Gras‐Le Guen et al. 2003). Notably, all the VLBW preterm babies developed severe forms of the infection, which led to their deaths. Meanwhile, 80% of infected full‐term neonates had a milder clinical presentation. (Gras‐Le Guen et al. 2003).

#### 3.6.3 Bacillus cereus

Only one study (Decousser et al. 2013) reported on *B. cereus* contamination of DHM. The two neonates infected by *B. cereus* in this report shared similar characteristics to those reported by Liao and Tsai (2021). They are all preterm infants with A birth weight ≤ 1500 g. However, the presentations differed in terms of severity. While Liao and Tsai (2021) reported a milder infection by the organism, the two patients described by Decousser et al. (2013) had severe intestinal infections with enterocolitis, which resulted in peritonitis in one of them. None of these cases was, however, associated with mortality. This may indicate that infection with *B. cereus* is less lethal in comparison with *C. sakazakii*. It should be considered, however, that fewer cases of *B. cereus* infection were reported, which makes it difficult to make insightful comparisons.

### 3.7 Infections Associated With Mixed Feeding

Finally, Teramoto et al. (2010) reported a case of severe infection from *C. sakazakii* of a premature infant (gestational age of 26 weeks and 3 days, 629 g*)*, leading to the death of a VLBW infant fed with MEBM, PIF, and milk additive. The authors, however, failed to conduct a culture of the food and the environment to determine the likely source of the contamination (Table 5).

**TABLE 5:** Mixed feeding‐associated infections in high‐income countries, 2000–2014.

Authors and country	Feed	Bacteria	Total cases	No colonized	No infected	No of deaths	Diagnosis of infected cases	M:F of infected cases	GA of infected cases (weeks + dys)	Birth category of infected cases	BW of infected cases (g)
Teramoto et al. (2010) Japan	MEBM + PIF + milk additive	*C. sakazakii*	1	0	1	1	Neonatal infection	F	26 + 3	Preterm	629
Subtotal			**1**	**0**	**1**	**1**					

BW, birth weight; GA, gestational age.

### 3.8 Sources of Pathogenic Contamination

#### 3.8.1 Maternal Expressed Breast Milk

Milk extraction materials and breast pump cleaning environments are considered the origin of MEBM contamination in this review. Many studies reported a recovery of the attributed pathogen from a privately owned breast pump (Bowen et al. 2017; Rettedal et al. 2012; Smith and Serke 2016; Sundararajan et al. 2018). In these cases, inadequate cleaning practices by parents, such as failure to sterilize pumps after use (McMullan et al. 2018; Smith and Serke 2016), storing of breast pumps and their parts in a moist state (Smith and Serke 2016), cleaning pumps only with baby wipes after use (Sundararajan et al. 2018), and soaking and rinsing of pumps without scrubbing (Bowen et al. 2017), were reported. Other identified sources include a milk bottle washing brush (Mizuno et al. 2021), the domestic kitchen sink and drain (Bowen et al. 2017; Sundararajan et al. 2018), and hospital sinks and drains (Rettedal et al. 2012).

#### 3.8.2 Powdered Infant Formula

The reports showed that PIF contamination can occur from the manufacturing stage or during reconstitution. This review revealed a recovery of the infection‐causing pathogen from opened tins of PIF (Brett et al. 2005; Himelright et al. 2002; Taylor et al. 2021), unopened cans (Brett et al. 2005; Himelright et al. 2002), and reconstituted feed, in cases where the opened tin of PIF tested negative (Bar‐Oz et al. 2001; Sánchez‐Carrillo et al. 2009). Further investigation to determine the source of the contaminant of prepared feeds pinpointed a shared hospital‐owned milk blender (Bar‐Oz et al. 2001) and dishwashers in the hospital's feeding bottle cleaning room (Sánchez‐Carrillo et al. 2009).

#### 3.8.3 Donor Human Milk

Although Bechmann et al. (2023) identified a culture‐positive stethoscope, it was not considered the possible origin of the infection, given that each patient had a dedicated stethoscope. Gras‐Le Guen et al. (2003) reported the milk bank pasteurizer and the hospital bottle warmer as the probable sources, while Decousser et al. (2013) identified a unique likely source of the pathogen—the nasogastric tube used in feeding the neonates, in a context where the DHM tested negative for the bacteria isolated from the infected case.

**TABLE 6:** Contaminant source investigation and findings of maternal expressed breast milk‐associated infections in high‐income countries, 2000–2014.

Authors	Exposure	Milk culture results	Result of environmental sampling	Confirmation of strain similarity with clinical isolates	Additional information considered
Bowen et al. (2017)	MEBM +DHM +fortifier	+ve	+ve personal breast pump +ve home kitchen sink drain	Confirmed	Mother failed to scrub the breast pump. The fortifier tested culture‐negative.
Christoph et al. (2011)	MEBM	+ve	Not reported	Not reported	Only the twins tested positive for *E. coli* in the NICU.
Liao and Tsai (2021)	MEBM	+ve	Not reported	Confirmed	Not reported.
McMullan et al. (2018)	MEBM	+ve	Not reported	Confirmed	History of using an unsterilized pump.
Mizuno et al. (2021)	MEBM	Not reported	+ve personal bottle washing brush	Confirmed	Not reported.
Ravisankar et al. (2014)	MEBM	−ve	Not reported	Not applicable due to −ve MEBM	No history of PIF or Fortifier use
Rettedal et al. (2012)	MEBM	+ve	+ve breast pump +ve hospital sink drain	Confirmed	Culture from all discharged cases before the index case admission tested negative.
Smith and Serke (2016)	MEBM	Cases 1, 2, 3 +ve	Case 1. +ve personal breast pump. Case 2 and 3. Not reported	Not reported	Case 1. Mother failed to sterilize the pump. Cases 1 &2 prolonged deterioration resolved only when MEBM was changed to DHM
Sundararajan et al. (2018)	MEBM	+ve	+ve personal breast pump +ve home sink, drain, and breast pump drying area	Confirmed	No history of PIF or fortifier ingestion. Mother rarely washed the pump with soap and water.
Widger et al. (2010)	MEBM	+ve	Not reported	Cases 1 and 2. Confirmed Case 3. Not reported	Case 3. MEBM and the clinical isolate have similar antibiotic susceptibility
Bar‐Oz et al. (2001)	PIF	+ve prepared feed ‐ve opened a tin of PIF	+ve hospital milk blender	Not reported	All cases were on exclusive PIF reconstituted using the same blender
Brett et al. 2005	PIF	+ve opened and unopened tins of PIF	−ve	Confirmed	+ve for a different strain of the pathogen
Himelright et al. 2002	PIF	+ve opened and unopened tins of PIF	−ve	Confirmed	All the cases were exposed to the PIF batch
Meeks et al. (2023)	PIF	Not reported	Not reported	Not reported	History of exclusive PIF feeding. History of earlier recall of the same batch of PIF by the manufacturer.
Sánchez‐Carrillo et al. (2009)	PIF	+ve reconstituted PIF	+ve hospital dishwasher in milk room	Confirmed	All positive samples showed similar antibiotic susceptibility.
Taylor et al. (2021)	PIF	Case 1. Not reported. Case 2. +ve opened a tin of PIF	Not reported	Not reported	Different strains from the two cases. Molecular analysis conducted by the Texas Public Health Dept. (Author not privy to report)
Bechmann et al. (2023)	DHM	+ve	+ve stethoscope	Confirmed	40% of affected neonates were fed from the pathogenic positive DHM, and the rest cases shared the same room with them
Decousser et al. (2013)	DHM	−ve	+ve milk in NG tube	−ve	Both cases share a similar strain of pathogen, which belongs to the same phylogenetic group and high virulence as that isolated from the NG tube.
Gras‐Le Guen et al. (2003)	DHM	Not reported	+ve Milk bank pasteurizer +ve hospital bottles warmer.	Confirmed	Not reported.
Teramoto et al. (2010)	MEBM + PIF + Milk additive	Not reported	Not reported	Not reported	Not reported

Abbreviations: +ve, positive; −ve, negative; DHM, donor human milk; MEBM, maternal expressed breast milk; NG tube, nasogastric tube; PIF, powdered infant formula.

### 3.9 Differences in the Number of Infections and Deaths

Overall, there is a remarkable difference in the number of infections and deaths between MEBM and the other methods of feeding. Although contaminated MEBM reported the highest total number of cases (71) compared with PIF and DHM, which reported 53 and 50, respectively, in terms of the actual number of infected infants, fewer cases were linked to MEBM (13) as against 23 and 18, respectively, under contaminated PIF and DHM categories. A similar picture is seen in the mortality figures. Only about 15% of the reported deaths were attributed to MEBM contamination; meanwhile, PIF and DHM contamination accounted for the bulk of the deaths (about 40% each), and 5% represents the death of the patient with mixed feeding. When PIF and DHM are compared, a significant difference does not seem to exist in the overall number of cases, the number of infants with systemic infections, or deaths. This may suggest a better outcome with MEBM.

## 4 Discussion

This review summarizes the evidence on infant infections and deaths associated with contaminated MEBM, PIF, and DHM in high‐resource countries in the last 25 years. In alignment with the objectives, it unraveled the difference in occurrences between these feeding options and identified the bacterial contaminants associated with these cases and the sources of the contaminants. The discussion will be organized in line with the objectives.

### 4.1 Differences in the Number of Infections and Deaths

A total of 175 cases of infant milk contamination‐related cases were identified. This demonstrates that the cases of systemic infections from contaminated MEBM and DHM are not as rare as previously reported (Blackshaw et al. 2021). It, however, revealed a lower number of cases of symptomatic infections and fatality with MEBM contamination compared with the other feeding alternatives and an unremarkable difference between PIF and DHM.

This finding agrees with a previous systematic review of studies from developed countries, even though the paper set of their review, contrary to the one used in this review, consisted only of observational studies (WHO 2011). Although reported with low to moderate certainty evidence, the author observed a reduction in mortality by 18% (95% CI, 7%–28%, based on four studies) and in severe infections by 60% (95% CI, 48%–69%, based on eight studies) in low birth weight and preterm infants fed with MEBM compared with PIF. Furthermore, the close number of cases and deaths associated with PIF and DHM observed in this analysis also corresponds with the findings of this previous study (WHO 2011). It is worth noting that the scope of infections examined by this author is all‐cause infection and not limited to those arising solely from contaminated MEBM, PIF, and DHM, as examined in this review, but they arrived at a similar conclusion. Strobel et al. (2022) and Blackshaw et al. (2021), nevertheless, reported a different finding. Strobel et al. (2022), in their study of the risk of severe infections and mortality between the three feeding practices, found no difference, even though their review equally consisted of only observational studies. Although the review had its strength in the huge number of participants that contributed to that conclusion (10,636 preterm neonates), a limitation of the review, acknowledged by the authors, which could provide a possible explanation for the difference in findings is the challenge that confounders were not addressed in the included studies, leading low‐ to very‐low‐certainty evidence (Strobel et al. 2022). On the other hand, Blackshaw et al. (2021) argued that in terms of protection from the risk of infection, DHM offers better outcomes compared with PIF. It must be reported that there is a general paucity of reviews examining infant infection and deaths arising specifically from infant milk contamination, and this presents limited opportunities to make comparisons with previous findings. Besides, the inconsistent findings highlight the need for more high‐quality studies on this subject.

Furthermore, this result appears to support the evidence that where direct breastfeeding cannot be carried out, such as in VLBW preterm infants or newborns separated from their mothers due to hospitalization, the use of MEBM is the next option (Meek and Noble 2022). An aspect of this result, though, should be interpreted with caution. While an insignificant difference was observed between PIF and DHM, it does not suggest that both are associated with the same overall clinical outcomes. This finding in this review is specific to two outcomes: infant infection and deaths associated with the ingestion of contaminated infant milk. Many studies agree that DHM is associated with less incidence of necrotizing enterocolitis compared with PIF (Boyd et al. 2007; Lucas and Cole 1990; McGuire and Anthony 2003), although findings pertaining to its edge over PIF in other outcomes, such as neonatal growth, have been inconsistent, and the available evidence is adjudged as low (Moreira‐Monteagudo et al. 2022; Pithia et al. 2024; Quigley et al. 2019). This, though, is outside the scope of this review.

### 4.2 Commonly Associated Pathogens

Although *Klebsiella, B. cereus*, and *S. aureus* were part of the contaminants linked to neonatal infection in MEBM and *C. botulinum* in PIF, this review identified *C. sakazakii* and *P. aeruginosa* as the two most implicated organisms that caused infections and deaths. For DHM, *B. cereus, S. marcescens*, and *P. aeruginosa* were identified. However, due to a scarcity of reports on DHM, the most common pathogen‐associated could not be identified.

Contrary to the findings of this review, Blackshaw et al. (2021) reported *Salmonella* species as the most common pathogen associated with infections and morbidity from PIF contamination. Meanwhile, a single case of *Salmonella* was not reported in the paper set for this review. The reason for this difference is unclear, as the authors extended their search to 2018, which is within the consideration of this review and equally focused on high‐resource countries. Moreover, previous publications that reported microbial contaminants in expressed breast milk have often identified *Staphylococcus* species as the commonest contaminant (Landers and Updegrove 2010; Novak et al. 2000; Thayagabalu et al. 2024). In reality, as revealed by this review, *C. sakazakii* appears to be the most reported in peer‐reviewed publications of clinical practice. This is consistent with Schanler et al.’s (2011) argument that isolating bacteria from random breast milk cultures is not a predictor of neonatal infection.

In this review, 85% of reported *C. sakazakii*‐associated infections were associated with PIF contamination, and 15% were linked to MEBM contamination. This reflects the findings of Jason (2012), in which 90% of neonatal *C. sakazakii* cases were related to PIF. Also, this review identified 5 deaths from 27 reported cases of *C. sakazakii*, about 1 in 5, which is consistent with the 17.7% reported by Blackshaw et al. (2021). Out of the 11 cases of *C. sakazakii* presented with full clinical details, nine were cases of meningitis with resulting severe brain pathology. Considering the highly invasive nature of this pathogen, *C. sakazakii* infections could be regarded as a case of one too many. Hence, there is a growing need to include infection with the pathogen among notifiable diseases, as there may be an underreporting. This seems to be the case in some states in the United States, as six out of nine case reports on *C. sakazakii* were from the United States, many of which were reported to the State Department of Health (Bowen et al. 2017; Himelright et al. 2002; Meeks et al. 2023; Sundararajan et al. 2018; Taylor et al. 2021). Generally, these findings emphasize the need to promote reporting of contaminated infant milk incidents to expose the actual figures and associated pathogens and improve the relevant policies and practices. *C. sakazakii* is a nonspore‐forming, Gram‐negative bacterium of the Enterobacteriaceae family, found in the environment. As a pathogen, it poses a great concern in the PIF manufacturing industry, owing to its ability to survive the desiccation of the PIF manufacturing process and the low moisture content of PIF, a condition that vegetative bacteria find quite challenging to survive (Lang et al. 2024). This ability has been associated with its ability to develop osmoprotectants and proteins that resist oxidation (Lang et al. 2024). Therefore, it is found in a wide range of food products, including dried foods (El‐Sharoud et al. 2009). It reportedly has a much higher tendency to contaminate infant milk production compared with *Salmonella* spp., as a study reported its prevalence in a group of milk manufacturing environments as 69%, whereas *Salmonella* was only 5.5% (Hayman et al. 2020). Besides, it is also known to form biofilms on stainless steel surfaces, which were found resistant to commonly used hospital disinfectants (Kim et al. 2007), feeding bottles, feeding tubes, and bottle screw covers, which makes decontamination very difficult and serves as a source of contamination for feeds subsequently prepared with such materials (Hurrell et al. 2009). Therefore, adequate cleaning practices must be implemented.

### 4.3 Sources of Pathogenic Contamination

#### 4.3.1 Extent of Contaminant Source Investigation

Remarkably, homogeneity is lacking in the extent of investigations conducted and the criteria used by the different authors to establish the source of the implicated organism (Table 6). On one end of the spectrum, some case reports were quite detailed in their investigations. They conducted a culture of the milk, the mother's home environment, the milk collection and preparation kit, and the hospital milk preparation rooms, and even went further to check for similarities in the molecular features of the strains of the bacteria recovered from suspected sources with the one cultured from the affected infant to confirm the source (Bowen et al. 2017; Decousser et al. 2013; Rettedal et al. 2012; Sundararajan et al. 2018). On the other end, some authors limited their source investigation to only the culture of the milk samples without molecular analysis (Christoph et al. 2011; Ravisankar et al. 2014; Taylor et al. 2021), and some reported no source investigation (Meeks et al. 2023; Teramoto et al. 2010). Admittedly, going the extra length to combine microbiological evidence from multiple sources appears to lend stronger evidence to the source of infection. Prima facie, this seems to question the validity of other case reports that lack such rigor. Nevertheless, some of the reports without extensive bacteriological proof of the source of contamination had additional information from patients' clinical history to support the source diagnosis (Christoph et al. 2011; Meeks et al. 2023; Ravisankar et al. 2014; Smith and Serke 2016). Therefore, the validity of each study is better judged on its own merits by summing all available information with the bacteriological evidence reported.

As revealed in this review, the milk collection and preparation kits and the environment where the milk is prepared largely remain the likely origin of the bacteria responsible for infant milk contamination. The breast pump hygiene practices by mothers reported in this review remain less than ideal. These reports were mainly from the NICUs, and milk storage was not identified as the main challenge. This finding supports the evidence from previous observations (Haiden et al. 2016; Peters et al. 2016). Unfortunately, this challenge continues despite the published guidelines on cleaning breast pumps and parts (BDA 2019; Centers for Disease Control and Prevention 2024). The BDA (2019) guidelines recognize the significant positive effect of combining good personal and milk handling hygiene, good quality water in rinsing breast pump parts and feeding utensils after washing, and adequate drying of all parts in reducing infant feed contamination. To mothers, these guidelines recommend the following hygiene practices: proper hand washing before expressing breast milk or feed preparation, PIF to be prepared for instant use and not stored for subsequent use, daily breast washing and a bra change to a clean one, the use of a dedicated wash basin for cleaning pump components and infant feeding utensils instead putting them in direct contact with the sink which has been identified in this review as a pathogen‐harboring point (Bowen et al. 2017; Rettedal et al. 2012; Sundararajan et al. 2018), disassembling pump parts after use, rinsing with water to remove the milk, thereafter, washing with warm soapy water, scrubbing, rinsing out the detergent with sterile water where the microbiological quality of available water cannot be ascertained, air‐drying or drying with clean paper towels and storing in a dry storage box. In addition, scrubbing brushes and the wash basin should be rinsed and air‐dried after use while ensuring that the milk preparation countertops, the surfaces of breast pumps, the tubes, the power buttons, and the dials are cleaned with disinfectant‐containing wipes (BDA 2019; Centers for Disease Control and Prevention 2024). Every 24 h, the steam sterilization method is encouraged. Concerning domestic refrigerator storage, the BDA guidelines emphasize collecting the expressed milk in hospital‐approved milk containers and placing it in a fridge, above the food compartments, at a temperature of 2–4°C for a maximum of 48 h. For hospital staff in charge of infant feeds, training policy should mandate safe feed materials handling training, and food hygiene qualification should be a requirement. Parents should be instructed to clean the shared electric pump after each use, while a dedicated breast pump should be provided for mothers with infections, who need to be decontaminated after every use. For storage, milk‐storing fridges or freezers should be dedicated solely for the purpose and not be utilized for storing foods and should be under lock with restricted access (BDA 2019). Following this recommendation is important because there is no policy mandating the pasteurization of the biological mother's expressed breastmilk (Red book 2006, as cited in Widger et al. (2010), but it requires great commitment and a change on the part of mothers (Haiden et al. 2016). Our finding of persistently hygienically suboptimal milk collection materials’ handling can be explained using the Health Belief Model (HBM), and this framework can equally be used to design interventions to address this critical challenge.

Therefore, clinicians need to assess mothers for possible barriers that might jeopardize clean milk collection and address identified challenges. When these are lacking, noncompliance with guidelines and the supply of contaminated MEBM will continue. Therefore, all the constructs of the HBM need to be addressed to improve adherence to recommendations on milk handling hygiene and quality milk collection. While this model is discussed here with particular reference to mothers and MEBM, the constructs apply to the handling of PIF and DHM preparation and feeding materials and to milk bank and hospital staff responsible for cleaning milk equipment (Peters et al. 2016).

### 4.4 Limitations and Strengths

The findings of this review are not without limitations. The article screening was conducted by one person, with queries resolved by a second reviewer. The review is strictly based on cases reported in peer‐reviewed articles to enhance rigor and accuracy, which leaves out cases that might appear in non‐peer‐reviewed studies. Given that half of the papers were of moderate to low quality evidence, there are limitations regarding the confidence in the conclusions. Moreover, the evidence considered is mainly from case reports, which are positioned at the lower level in the hierarchy of evidence (Burns et al. 2011). RCTs were not found for this review, as the nature of the question makes it impractical. The few observational studies included in this review reported outbreaks. However, single cases and outbreaks are required to answer the research question. Therefore, findings from case reports, undeniably, add to the existing body of knowledge on the subject and provide a hypothesis for further higher‐level evidence studies (Burns et al. 2011). Moreover, the review demonstrated strength in several areas. First, the literature search was extensive, as it combined keywords, MeSH, and Emtree search terms using the largest databases to capture as much relevant literature as possible. Second, the methodology of study selection is transparently documented and reproducible. Third, considering that the quality of a review is as good as the quality of the papers it contains (Powell and Koelemay 2022), the papers included in this review consist mostly of those with high‐ to moderate‐quality evidence, as many of the authors eliminated confounding factors. Furthermore, this is the first scoping review to compare the number of cases of infant infections and deaths in strict relation to bacterial contaminated MEBM, PIF, and DHM.

## 5 Conclusions and Recommendations

Infant mortality remains a significant public health issue across the globe. The right nutrition is crucial to improve survival; breastfeeding has been acknowledged as the gold standard. When direct breastfeeding is not feasible, MEBM, DHM, and PIF are the available alternatives. However, these feeding alternatives can be contaminated with bacteria, which could lead to severe systemic infections and death. This review examined 25 years from peer‐reviewed publications of infant infections and deaths associated with contaminated breastfeeding alternatives within high‐resource countries and identified the organisms responsible and their sources to inform improvement in infant feeding practices.

A combination of an advanced database and a manual search of reference lists yielded the papers on which this result is premised. The result suggests that MEBM is the least associated with infection and death, and there is no significant difference between DHM and PIF when compared based on these two outcomes. Peters et al. (2016) indicated that pasteurization of MEBM is unnecessary. Furthermore, *C. sakazakii* and *P. aeruginosa* were the commonly peer‐reviewed reported organisms in neonatal infection cases consequent to contaminated PIF and MEBM in this review. Concerning the sources of bacterial contaminants, besides the intrinsic contamination of PIF, improperly cleaned milk handling materials and milk preparation areas have contributed hugely to the contamination of these infant feeds. Winning the commitment of mothers and hospital staff is crucial to record progress in this area.

Encouraging the collection of microbiologically safe milk through methods like MEBM is essential when breastfeeding is not feasible. Despite the reduction in immune‐protective properties of DHM caused by pasteurization (Ames et al. 2023), mandatory routine pasteurization should be considered to enhance safety. Additionally, given the virulence of *C. sakazakii* and its affinity for cerebral tissue, it is crucial to include this pathogen on the list of notifiable diseases. Adopting the HBM framework in educating mothers and hospital staff about milk‐handling hygiene could foster behavioral changes, as this model has proven effective in other domains (Razmara et al. 2018; Roditis et al. 2020). Moreover, clinicians must consider contaminant sources beyond hospital environments, extending investigations to include milk collection, pump cleaning, and storage practices in mothers' homes to ensure refrigeration at 4°C or below. While challenging, this comprehensive approach would help secure hygienic milk‐handling practices for infants even after discharge.

Future research should examine the risk of infection in infants caused by biofilm formation in enteral feeding tubes used for continuous milk administration. This work would guide the development of standardized protocols for the frequency of tube replacement in NICUs, an area beyond the scope of this review. Since there is most probably a significant underreporting of infant food contamination incidents, this review underscores the importance of improving reporting practices to address the lack of studies in this field. Authors also face challenges in differentiating neonatal infections caused by extrinsically contaminated milk from those arising from MTCT, compounded by inconsistent reporting criteria. Establishing standardized criteria, such as those proposed by Lawrence and Lawrence (2004) for MTCT, is essential for accurately documenting infections linked to milk contamination.

## Nomenclature

MEBM: maternal expressed Breast Milk
PIF: powdered infant formula
DHM: donor human milk
NICU: neonatal intensive care unit
VLBW: very low birth weight
MTCT: mother‐to‐child transmission
GA: gestational age
NR: not reported
BW: birth weight
NA: not applicable
NFD: no full detail
NG tube: nasogastric tube
OG tube: orogastric tube
FB: feeding bottle
MRSA: methicillin‐resistant *Staphylococcus aureus*
ESBL: extended‐spectrum beta‐lactamase
SIIE: severe intestinal infection and enterocolitis
SIIE+P: severe intestinal infection, enterocolitis with intestinal perforation

## Author Contributions

**Chelsea S. Amenah–James**: conceptualization, investigation, writing – original draft, writing – review and editing, methodology, data curation, visualization, project administration. **Ellen W. Evans**: conceptualization, writing – review and editing, methodology, software, supervision. **Sophia Komninou**: conceptualization, writing – review and editing, methodology, supervision, resources, project administration.

## Conflicts of Interest

All authors declare no conflicts of interest.

## Supporting information




**Supporting Appendix A**: crf370282‐sup‐0001‐Appendix‐A.docx

**Supporting Appendix B**: crf370282‐sup‐0002‐Appendix‐B.docx

**Supporting Appendix C**: crf370282‐sup‐0003‐Appendix‐C.docx

**Supporting Appendix D**: crf370282‐sup‐0004‐Appendix‐D.docx

## Data Availability

All search terms and critical appraisal information are submitted as Supporting Information.
