# Supplementary material for: Foodborne Infections and Mortality Associated With Expressed Breastmilk, Donated Breastmilk, and Infant Formula in High‐Income Countries: A Scoping Review of Peer‐Reviewed Evidence Cases
Source: Compr Rev Food Sci Food Saf. 2025 Sep 19;24(5):e70282. doi: 10.1111/1541-4337.70282 (PMC12447545; doi:10.1111/1541-4337.70282)
Supplement: Supplementary file 1 — Supporting Appendix A: crf370282‐sup‐0001‐Appendix‐A.docx [file CRF3-24-e70282-s004.docx]

Appendix A

Search Strategy on Medline

| Concepts | Abstract and Title Phrase Search Terms | Mesh terms |
| --- | --- | --- |
| Population | AB ( newborn* or infant* or preterm* or pre-term* or prematur* or neonat* or “low-birth-weight” or “low birth weight" or bab* or child* ) OR TI ( newborn* or infant* or preterm* or pre-term* or prematur* or neonat* or “low-birth-weight” or “low birth weight" or bab* or child* ) | (MH "Infant+") |
| Exposure 1 MEBM | AB ( “expressed breastmilk” or “expressed breast milk” or “expressed human milk” or “expressed mother’s milk” or EBM or “breastmilk expression” or “human milk provision” or “milk expression” or “hand expression” or “manual expression” or “breast pumping” or “hands-on pumping” or “expressed milk” ) OR TI ( “expressed breastmilk” or “expressed breast milk” or “expressed human milk” or “expressed mother’s milk” or EBM or “breastmilk expression” or “human milk provision” or “milk expression” or “hand expression” or “manual expression” or “breast pumping” or “hands-on pumping” or “expressed milk” ) | (MH "Breast Milk Expression") |
| Exposure 2 PIF | AB ( “breastmilk substitute*” or PIF or “infant formula” or “milk substitute*” or “replacement formula” or “preterm formula” or “breast milk substitute*” ) OR TI ( “breastmilk substitute*” or PIF or “infant formula” or “milk substitute*” or “replacement formula” or “preterm formula” or “breast milk substitute*” ) | (MH "Infant Food+") |
| Exposure 3 DHM | AB (“donor milk” or “donated human milk” or “banked milk” or pasteuri* or “banked human milk” ) OR TI ( “donor milk” or “donated human milk” or “banked milk” or pasteuri* or “banked human milk” ) | MH "Milk Banks") OR (MH "Milk, Human") OR (MH "Pasteurization") |
| Outcome | AB ( sepsis or bacteremia or “septic shock” or septicemia or septiceamia or infection* or “necrotizing enterocolitis” or “necrotising enterocolitis” or morbidity or mortality or salmonel* or cronobacter or enterobacter or “group B streptococc*” or listeri* or monocytogenes or MRSA or “Escherichia coli” or “E. coli” or “Serratia marcescens” or “bacillus cereus” or “Staphylococcus aureus” or cytomegalovirus or HCMV or pathogen* ) OR TI ( sepsis or bacteremia or “septic shock” or septicemia or septiceamia or infection* or “necrotizing enterocolitis” or “necrotising enterocolitis” or morbidity or mortality or salmonel* or cronobacter or enterobacter or “group B streptococc*” or listeri* or monocytogenes or MRSA or “Escherichia coli” or “E. coli” or “Serratia marcescens” or “bacillus cereus” or “Staphylococcus aureus” or cytomegalovirus or HCMV or pathogen*) | (MH "Sepsis") OR (MH "Bacteremia+") OR (MH "Shock, Septic") OR (MH "Neonatal Sepsis") OR (MH "Gram-Positive Bacterial Infections+") OR (MH "Listeriosis+") OR (MH "Staphylococcal Infections") OR (MH "Streptococcal Infections") OR (MH "Staphylococcal Food Poisoning") OR  (MH "Gram-Negative Bacterial Infections") OR (MH "Enterobacteriaceae Infections") OR (MH "Serratia Infections") OR (MH "Salmonella Infections+") OR (MH "Klebsiella Infections") OR (MH "Escherichia coli Infections+") OR (MH "Enterocolitis, Necrotizing") OR (MH "Cytomegalovirus Infections") |
| Study design | AB ( “Case stud*” or “Case report*” or RCT or trial* or cohort or “Observational stud*” or “quantitative method*” or “quantitative stud*” or “quantitative research” ) OR TI ( “Case stud*” or “Case report*” or RCT or trial* or cohort or “Observational stud*” or “quantitative method*” or “quantitative stud*” or “quantitative research” ) | (MH "Case Reports as Topic") OR (MH "Randomized Controlled Trials as Topic") OR (MH "Cohort Studies+") OR (MH "Cross-Sectional Studies") OR (MH "Observational Studies as Topic") OR (MH "Single-Case Studies as Topic") |

Search Strategy on Embase

| Concepts | Title, Abstract, Keyword Search Terms | Emtree Subject headings |
| --- | --- | --- |
| Population | (Newborn* OR infant* OR preterm* OR pre-term* OR prematur* or neonat* OR “low-birth-weight” OR “low birth weight" OR bab* OR child*):ti,ab,kw | 'Infant'/exp  'Prematurity'/exp  'Low birth weight'/exp  'child'/de |
| Exposure 1 MEBM | (“Expressed breastmilk” OR “expressed breast milk” OR “expressed human milk” OR “expressed mother* milk” OR EBM OR “breastmilk expression” OR “human milk provision” OR “milk expression” OR “hand expression” OR “manual expression” OR “breast pumping” OR “hands-on pumping” OR “expressed milk”):ti,ab,kw | 'Breast milk expression'/de  'Pump'/exp  'Expressed breast milk'/exp |
| Exposure 2  PIF | (“breastmilk substitute*” OR PIF OR “infant formula” OR “milk substitute*” OR “replacement formula” OR “preterm formula” OR “breast milk substitute*”):ti,ab,kw | 'Milk substitute'/exp  'Baby food'/exp |
| Exposure 3  DHM | (“donor milk” OR “donated human milk” OR “banked milk” OR pasteuri* OR “banked human milk”):ti,ab,kw | 'Donor milk'/de  'Milk bank'/de  'pasteurization'/de |
| Outcomes | (sepsis OR bacteremia OR “septic shock” OR septicemia OR septiceamia or infection* OR “necrotizing enterocolitis” OR “necrotising enterocolitis” or morbidity or mortality or salmonel* OR cronobacter OR enterobacter OR “group B streptococc*” OR listeri* OR monocytogenes OR MRSA OR “Escherichia coli” OR “E. coli” OR “Serratia marcescens” OR “bacillus cereus” OR “Staphylococcus aureus” OR cytomegalovirus OR HCMV OR pathogen*):ti,ab,kw | 'sepsis'/exp  'septic shock'/exp  'necrotizing enterocolitis'/de  'salmonella'/exp  'Cronobacter sakazakii'/de  'Enterobacter'/de  'Group B streptococcal infection'/exp  'listeria'/de  'Listeria monocytogenes'/exp  'listeriosis'/exp  'Methicillin resistant staphylococcus aureus'/de  'Escherichia coli'/de  'Serratia marcescens'/de  'Bacillus cereus'/de  'Staphylococcus aureus'/de  'Cytomegalovirus infection'/de  'cytomegalovirus'/de  'Breastfeeding transmission'/de  'Gram positive infection'/de  'Streptococcus infection'/de  'Staphylococcus aureus'/de  'Staphylococcus epidermidis'/de  'Staphylococcal food poisoning'/de  'gram-negative infection'/de |
| Study Types | (“Case stud*” or “Case report*” or RCT or trial* or cohort or “Observational stud*” or “quantitative method*” or “quantitative stud*” or “quantitative research”):ti,ab,kw | 'case study'/exp  'case report'/de  'randomized controlled trial'/de  'cohort analysis'/de  'cross-sectional study'/de  'observational study'/de  'quantitative study'/de  'quantitative research'/de |
