# Supplementary material for: Foodborne Infections and Mortality Associated With Expressed Breastmilk, Donated Breastmilk, and Infant Formula in High‐Income Countries: A Scoping Review of Peer‐Reviewed Evidence Cases
Source: Compr Rev Food Sci Food Saf. 2025 Sep 19;24(5):e70282. doi: 10.1111/1541-4337.70282 (PMC12447545; doi:10.1111/1541-4337.70282)
Supplement: Supplementary file 2 — Supporting Appendix B: crf370282‐sup‐0002‐Appendix‐B.docx [file CRF3-24-e70282-s001.docx]

Appendix B

**JBI Critical Appraisal for the Cohort Studies**

|  |  |  |  |  |  |  |  |  |  |  |  |  |
| --- | --- | --- | --- | --- | --- | --- | --- | --- | --- | --- | --- | --- |
| Study author | Q1 | Q2 | Q3 | Q4 | Q5 | Q6 | Q7 | Q8 | Q9 | Q10 | Q11 | Total |
| Himelright et. al | √ | - | √ | √ | √ | √ | √ | √ | √ | √ | N/A | 9/10 |
|  | | | | | | | | | | | |  |
| YES   √  NO X UNCLEAR ----  NOT APPLICABLE N/A    Q1. Were the two groups similar and recruited from the same population?  Q2. Were the exposures measured similarly to assign people to both exposed and unexposed groups?  Q3. Was the exposure measured in a valid and reliable way?  Q4. Were confounding factors identified?  Q5. Were strategies to deal with confounding factors stated?  Q6. Were the groups/participants free of the outcome at the start of the study (or at the moment of exposure)?  Q7. Were the outcomes measured in a valid and reliable way?  Q8. Was the follow-up time reported and sufficient to be long enough for outcomes to occur?  Q9. Was follow-up complete, and if not, were the reasons to loss to follow-up described and explored?  Q10. Were strategies to address incomplete follow-up utilized?  Q11. Was appropriate statistical analysis used? | | | | | | | | | | | |  |
