# Supplementary material for: Foodborne Infections and Mortality Associated With Expressed Breastmilk, Donated Breastmilk, and Infant Formula in High‐Income Countries: A Scoping Review of Peer‐Reviewed Evidence Cases
Source: Compr Rev Food Sci Food Saf. 2025 Sep 19;24(5):e70282. doi: 10.1111/1541-4337.70282 (PMC12447545; doi:10.1111/1541-4337.70282)
Supplement: Supplementary file 3 — Supporting Appendix C: crf370282‐sup‐0003‐Appendix‐C.docx [file CRF3-24-e70282-s003.docx]

Appendix C

**JBI Critical Appraisal for the Case-Control Studies**

|  |  |  |  |  |  |  |  |  |  |  |  |  |
| --- | --- | --- | --- | --- | --- | --- | --- | --- | --- | --- | --- | --- |
| Author | Q1 | Q2 | Q3 | Q4 | Q5 | Q6 | Q7 | Q8 | Q9 | Q10 | Q11 | Total |
| Bechmann et al | √ | √ | √ | √ | √ | √ √ | N/A | N/A | √ | √ | - | 8/9 |
|  | | | | | | | | | | | |  |
| YES   √  NO X CAN’T TELL ----  NOT APPLICABLE N/A    Q1. Did the study address a clearly focused issue?  Q2. Did the authors use an appropriate method to answer their question?  Q3. Were the cases recruited in an acceptable way?  Q4. Were the controls selected in an acceptable way?  Q5. Was the exposure accurately measured to minimise bias?  Q6a. Aside from the experimental intervention, were the groups treated equally?  Q6b. Have the authors taken account of the potential confounding factors in the design and/or in their analysis?  Q7. How large was the treatment effect?  Q8. How precise was the estimate of the treatment effect?  Q9. Do you believe the results?  Q10. Can the results be applied to the local population?  Q11. Do the results of this study fit with other available evidence? | | | | | | | | | | | |  |
