# Supplementary material for: Foodborne Infections and Mortality Associated With Expressed Breastmilk, Donated Breastmilk, and Infant Formula in High‐Income Countries: A Scoping Review of Peer‐Reviewed Evidence Cases
Source: Compr Rev Food Sci Food Saf. 2025 Sep 19;24(5):e70282. doi: 10.1111/1541-4337.70282 (PMC12447545; doi:10.1111/1541-4337.70282)
Supplement: Supplementary file 4 — Supporting Appendix D: crf370282‐sup‐0004‐Appendix‐D.docx [file CRF3-24-e70282-s002.docx]

Appendix D

**Published modified Newcastle-Ottawa Score for Case Reports**

|  |  |  |  |  |  |  |  |  |  |
| --- | --- | --- | --- | --- | --- | --- | --- | --- | --- |
| Author | Q1 | Q2 | Q3 | Q4 | Q5 | Q6 | Q7 | Q8 | Total out of 6 |
| Bar-Oz et al | √ | √ | √ | √ | N/A | N/A | √ | √ | 6 |
| Bowen et al | √ | √ | √ | √ | N/A | N/A | √ | √ | 6 |
| Brett et al | √ | √ | √ | √ | N/A | N/A | √ | √ | 6 |
| Christoph et al | √ | √ | √ | X | N/A | N/A | √ | √ | 5 |
| Decousser et al | √ | √ | √ | √ | N/A | N/A | v | √ | 6 |
| Gras-Le Guen et al | √ | √ | √ | √ | N/A | N/A | √ | √ | 6 |
| Mcmullan et al | √ | √ | √ | x | N/A | N/A | √ | √ | 5 |
| Meeks et al | √ | √ | √ | x | N/A | N/A | √ | X | 4 |
| Ravisankar et al | √ | √ | √ | x | N/A | N/A | √ | √ | 5 |
| Rettedal et al | √ | √ | √ | √ | N/A | N/A | √ | √ | 6 |
| Smith & Serke | √ | √ | √ | x | N/A | N/A | √ | X | 4 |
| Sundararajan et al | √ | √ | √ | √ | N/A | N/A | √ | X | 5 |
| Widger et al | √ | √ | √ | x | N/A | N/A | √ | X | 4 |
| Taylor et al | √ | √ | √ | x | N/A | N/A | √ | x | 4 |
| Mizuno et al | √ | √ | √ | √ | N/A | N/A | √ | √ | 6 |
| Liao &Tsai | √ | √ | √ | x | N/A | N/A | √ | √ | 5 |
| Sanchez-Carrillo et al | √ | √ | √ | √ | N/A | N/A | √ | √ | 6 |
| Teramoto et al | √ | x | √ | x | N/A | N/A | √ | X | 3 |
| YES   √  NO X UNCLEAR ----  NOT APPLICABLE N/A  Q1. Does the patient(s) represent(s) the whole experience of the investigator (centre) or is the selection method unclear to the extent that other patients with similar presentation may not have been reported?  Q2. Was the exposure adequately ascertained?  Q3. Was the outcome adequately ascertained?  Q4. Were other alternative causes that may explain the observation ruled out?  Q5. Was there a challenge/rechallenge phenomenon?  Q6. Was there a dose–response effect?  Q7. Was follow-up long enough for outcomes to occur?  Q8. Is the case(s) described with sufficient details to allow other investigators to replicate the research or to allow practitioners make inferences related to their own practice? | | | | | | | | | |
